# Polymer-Mediated Delivery of siRNAs to Hepatocellular Carcinoma: Variables Affecting Specificity and Effectiveness

**DOI:** 10.3390/molecules23040777

**Published:** 2018-03-28

**Authors:** Rossella Farra, Francesco Musiani, Francesca Perrone, Maja Čemažar, Urška Kamenšek, Federica Tonon, Michela Abrami, Aleš Ručigaj, Mario Grassi, Gabriele Pozzato, Deborah Bonazza, Fabrizio Zanconati, Giancarlo Forte, Maguie El Boustani, Lucia Scarabel, Marica Garziera, Concetta Russo Spena, Lucia De Stefano, Barbara Salis, Giuseppe Toffoli, Flavio Rizzolio, Gabriele Grassi, Barbara Dapas

**Affiliations:** 1 Department of Engineering and Architecture, University of Trieste, Via Alfonso Valerio, 6/A, I-34127 Trieste, Italy; rfarra@units.it (R.F.); Michela.Abrami@dia.units.it (M.A.); mario.grassi@dia.units.it (M.G.); 2 Laboratory of Bioinorganic Chemistry, Department of Pharmacy and Biotechnology, University of Bologna, I-40127 Bologna, Italy; Francesco.musiani@unibo.it; 3 Department of Life Sciences, Cattinara University Hospital, Trieste University, Strada di Fiume 447, I-34149 Trieste, Italy; Francesca.Perrone@phd.units.it (F.P.); effetonon@gmail.com (F.T.); bdapas@units.it (B.D.); 4 Department of Experimental Oncology, Institute of Oncology, Ljubljana, Zaloska 2, SI-1000 Ljubljana, Slovenia; MCemazar@onko-i.si (M.Č.); UKamensek@onko-i.si (U.K.); 5 Faculty of Health Sciences, University of Primorska, Polje 42, SI-6310 Izola, Slovenia; 6 Faculty of Chemistry and Chemical Technology, University of Ljubljana, Večna pot 113, SI-1000 Ljubljana, Slovenia; Ales.Rucigaj@fkkt.uni-lj.si; 7 Department of “Scienze Mediche, Chirurgiche e della Salute”, University of Trieste, Cattinara Hospital, Strada di Fiume 447, I-34149 Trieste, Italy; G.POZZATO@fmc.units.it (G.P.); deborah.bonazza@asuits.sanita.fvg.it (D.B.); fabrizio.zanconati@asuits.sanita.fvg.it (F.Z.); 8 Center for Translational Medicine (CTM), International Clinical Research Center (ICRC), St. Anne’s University Hospital, Studenstka 6, 656 91 Brno, Czech Republic; giancarlo.forte@fnusa.cz; 9 Experimental and Clinical Pharmacology Unit, C.R.O.-National Cancer Institute, via Franco Gallini 2, I-33081 Aviano (PN), Italy; elboustanymaguie@gmail.com (M.E.B.); lucia.scarabel@cro.it (L.S.); mgarziera@cro.it (M.G.); concettarussospena@gmail.com (C.R.S.); lucia.destefano077@gmail.com (L.D.S.); salis.barbara@libero.it (B.S.); gtoffoli@cro.it (G.T.); flavio.rizzolio@unive.it (F.R.); 10 Doctoral School in Molecular Biomedicine, University of Trieste, 34100 Trieste, Italy; 11 Doctoral School in Chemistry, University of Trieste, 34100 Trieste, Italy; 12 Department of Molecular Sciences and Nanosystems, Ca’ Foscari University, via Torino 155, I-30172 Mestre (Venezia), Italy

**Keywords:** optimized drug delivery, siRNA, HCC.

## Abstract

Despite the advances in anticancer therapies, their effectiveness for many human tumors is still far from being optimal. Significant improvements in treatment efficacy can come from the enhancement of drug specificity. This goal may be achieved by combining the use of therapeutic molecules with tumor specific effects and delivery carriers with tumor targeting ability. In this regard, nucleic acid-based drug (NABD) and particularly small interfering RNAs (siRNAs), are attractive molecules due to the possibility to be engineered to target specific tumor genes. On the other hand, polymeric-based delivery systems are emerging as versatile carriers to generate tumor-targeted delivery systems. Here we will focus on the most recent findings in the selection of siRNA/polymeric targeted delivery systems for hepatocellular carcinoma (HCC), a human tumor for which currently available therapeutic approaches are poorly effective. In addition, we will discuss the most attracting and, in our opinion, promising siRNA-polymer combinations for HCC in relation to the biological features of HCC tissue. Attention will be also put on the mathematical description of the mechanisms ruling siRNA-carrier delivery, this being an important aspect to improve effectiveness reducing the experimental work.

## 1. Introduction

Antisense oligonucleotides, triplex forming-oligo-nucleotides, ribozymes, DNAzymes, antisense oligonucleotides, aptamers, long non-coding RNAs, siRNAs and miRNAs belong to an attractive class of molecules collectively named nucleic acid-based drugs (NABDs) [1,2,3,4,5,6]. Despite the different mechanisms of actions, all these molecules can interact, in a sequence-related fashion, with the target that, depending on the different NABDs, can be represented by a nucleic acid or a protein. Because of the ability to interact virtually with all biological molecules in the cells, NABDs can regulate the pathological functions of aberrantly expressed/functioning biological molecules. Thus, NABDs have a great therapeutic potential. Nowadays, among NABDs, siRNAs (small interfering RNA) are the molecules mostly investigated as potential therapeutics. In this regard, many hopes are placed on siRNAs to combat the second cause of death in the industrialize Countries, i.e., tumors. Despite recent therapeutic improvements, for many human tumors including hepatocellular carcinoma (HCC), effective therapeutic approaches are not available.

In this review, we will concentrate on the description of the therapeutic potential of siRNAs and related delivery systems against HCC. After a brief description of HCC (Section 1.1) and siRNA (Section 1.2) features, we will summarize the barriers a siRNA has to overcome before reaching the target (Section 2). Then, we will describe the possible solutions to minimize siRNA delivery difficulties (Section 3) putting particular emphasis on the specific HCC related delivery issues (Section 4). Finally, we will describe some experimental applications of siRNA/delivery systems to HCC (Section 5).

### 1.1. HCC

HCC is the most frequent primary malignant neoplasm of the liver and one of the most lethal human cancer types [7]. HCC is currently the second cause of cancer death and the sixth most commonly diagnosed cancer worldwide [8]. HCC prognosis is very poor with a 5-year overall survival rate estimated at less than 12% [9]. Global variations in incidence rates of this cancer closely reflect the differences in HCC risk factors distribution among developed and developing countries [10]. Alcoholic liver disease (ALD) which can progress to cirrhosis, hepatitis C virus (HCV) infection and non-alcoholic fatty liver diseases (NAFLD) are common risk factors in Europe, Northern America and Japan. ALD and HCV infection, either alone or in combination, are responsible for more than two-thirds of all HCC cases in the Western world [11]. Regardless of the etiological cause, cirrhosis is the strongest predisposing factor being associated with the development of HCC [12]. In Asia and sub-Saharan Africa, exposure to dietary aflatoxin B1 associated with chronic hepatitis B virus (HBV) or HCV infections, is an important risk factor for the observed high rate of HCC [13,14]. Others risk factors for HCC development are the use of tobacco, autoimmune hepatitis, alpha-1 antitrypsin deficiency and hereditary hemochromatosis [13].

HCC, characterized by aberrant and exuberant angiogenesis with anomalous cell cycle control and evasion from apoptosis [15], displays high resistance to chemotherapy and radiation treatments [16,17]. Surgical resection, transplantation and ablation are treatments restricted to very early stages of the disease [18,19]. The first line systemic chemotherapy approved by FDA is based on the use of Sorafenib. This is an oral multi-kinase inhibitor blocking Raf/MEK/ERK pathway and other extracellular receptor tyrosine kinases. Sorafenib can also be administered via trans-arterial chemoembolization (TACE) [20,21]. TACE involves the combination of the selective injection through the hepatic artery of antineoplastic agents such as sorafenib with the selective obstruction of tumor feeding vessels. However, sorafenib clinical advantage is modest, prolonging both relapse-free survival and overall survival by only 2–3 months with a response rate of less than 5% [17,22].

### 1.2. siRNA

**Figure 1:**
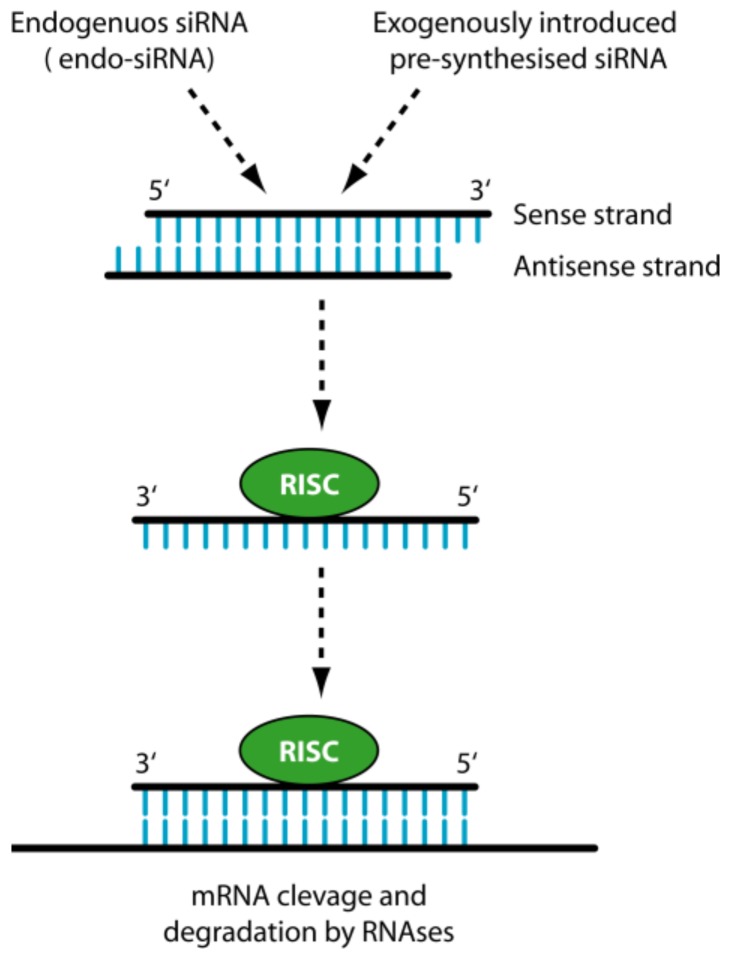
siRNA mechanism of action. The antisense strand of the siRNA is up-loaded by the catalytic protein complex RISC (RNA-induced silencing complex), while the sense strand is discarded. The antisense strand drives RISC to a target complementary mRNA, resulting in the specific RISC-mediated cleavage and subsequent degradation by cellular RNAses.

siRNAs are short double-stranded RNA molecules approximately 22 nucleotides long. They can be of intracellular (endo-siRNA) or exogenous origin, being in this last case generated from invasive nucleic acids such as viruses and transposons [23] or introduced for research purposes. With regard to their mechanism of action (Figure 1), of the two filaments forming siRNAs, one (antisense strand) is up-taken by a cellular protein complex termed RISC (RNA-induced silencing complex), while the other (sense strand) is not up-taken by RISC and is discarded. The antisense strand drives RISC to a target RNA via a perfect sequence complementarity to the target RNA. This results in the RISC-mediated degradation of the target RNA with the consequent down-regulation of gene expression. siRNA can be chemically generated and effectively used to target gene mRNA causing disease as shown by many works [3,24,25,26,27,28,29]. 

## 2. The Delivery Problems of siRNAs

**Figure 2:**
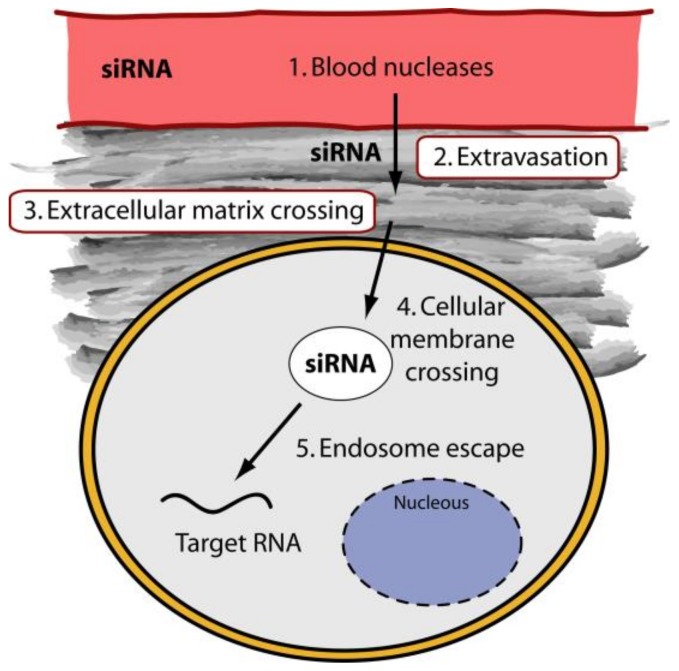
Biological barriers to siRNA delivery. For systemically released siRNAs the first obstacle is represented by blood nucleases, which can induce their degradation. The siRNAs have then problem to cross the vessel wall (extravasation) then the ECM (Extra Cellular Matrix) and finally the cell membrane. Once into the cell, siRNAs have to evade from endosome.

Despite the great therapeutic potential of siRNAs, their practical use is limited by the chemical nature that does not allow the administration in the naked form. When released systemically, siRNAs encounter blood nucleases, which can induce their rapid degradation (Figure 2). Additionally, naked siRNAs tend to be eliminated by the phagocytic system, by kidney filtration [30] and, depending on the sequence, to activate the innate immunity [31]. The fraction of siRNAs that escapes the above obstacles has the problem to cross the vessel wall (extravasation) and the cellular membrane. Cell membrane crossing is hindered by the electrostatic repulsion between the negatively charged phosphate groups present in siRNAs and the negatively charged surface of cellular membranes. In addition, the hydrophilic nature of siRNA obstacles the crossing of the hydrophobic layer of the cell membranes. The fraction of siRNA escaping the above obstacles has to face the problem of cytosolic nucleases that can further reduce the number of active molecules. Finally, proper cellular trafficking, i.e., endosomal escape [32], has to be accomplished. If siRNAs are sequestered into endosomes, they do not have the possibility to get in contact with their targets, thus drastically impairing if not abolishing the biological effect(s). Thus, it is clear that if administered as naked molecules, siRNAs have no possibility to exert any therapeutic function. 

## 3. General Strategies to Optimize siRNA Delivery 

Two main strategies are commonly followed to minimize the delivery obstacles of exogenously synthesized siRNAs. One possibility consists of the chemical modification of siRNA structure to make it resistant to degradation [4]. The other possibility is to complex siRNA with synthetic vectors able to bind, protect and allow the delivery to the target cells [4]. In this way, a nanostructured system can permit to increase tumor accumulation thus improving siRNA efficacy [33,34,35,36]. Often the two strategies are adopted together even if some chemical modifications may impair siRNA effectiveness. Thus, caution should be taken in the use of siRNA chemical modification(s). Here we will concentrate on the siRNA complexation with synthetic vectors [6,37,38,39,40] and in particular on polymeric-based vectors. These vectors are commonly used as siRNA delivery materials because of their versatility, biocompatibility and low cost. Polymers can provide siRNA protection against nucleases, allow ECM and cell membrane crossing and, when equipped with smart moieties, direct the siRNA to the target cells. A general description of the most commonly used polymers is reported below.

### 3.1. Polymers for siRNA Delivery

Polymers have been used in siRNA delivery due to: (1) the relatively low costs of production/isolation, (2) the possibility to undergo a wide range of chemical modifications that allow the optimization for the specific application, (3) the fact that they are biocompatible, biodegradable and have low immunogenic properties. Most commonly used polymers for siRNA delivery to HCC are Chitosan (CH), Inulin, PEI (Polyethylenimine), PEG (Polyethilenglycole), Polycaprolactone (PCL), and cyclodextrin (CD). Whereas CH and Inulin are natural polymers, PEI, PEG, PCL and CD, are of synthetic origin. Often these polymers are used in combination with each other or even with other different molecules to improve delivery properties.

The high plasticity of the molecular structure of polymers is probably at the base of their success as delivery systems for different drugs including, but not limited to, siRNAs. A relevant variable to be considered when developing siRNA-polymers complexes deals with the net superficial charge of the particles. Both anionic and cationic complexes usually display proper solubility and stability in the physiological environment. However, strongly anionic complexes can have difficulties in transfecting cells in virtue of the electrostatic repulsion with the negatively charged cell membrane. Excessively strong cationic complexes bind greedily to cell membrane due to potent electrostatic interactions; this, however, can lead to non-specific cellular uptake [41] and cause membrane disruption with consequent cell death. Thus, the surface electric charge has to be finely tuned to have optimal transfection with low unspecific cytotoxicity.

**Figure 3:**
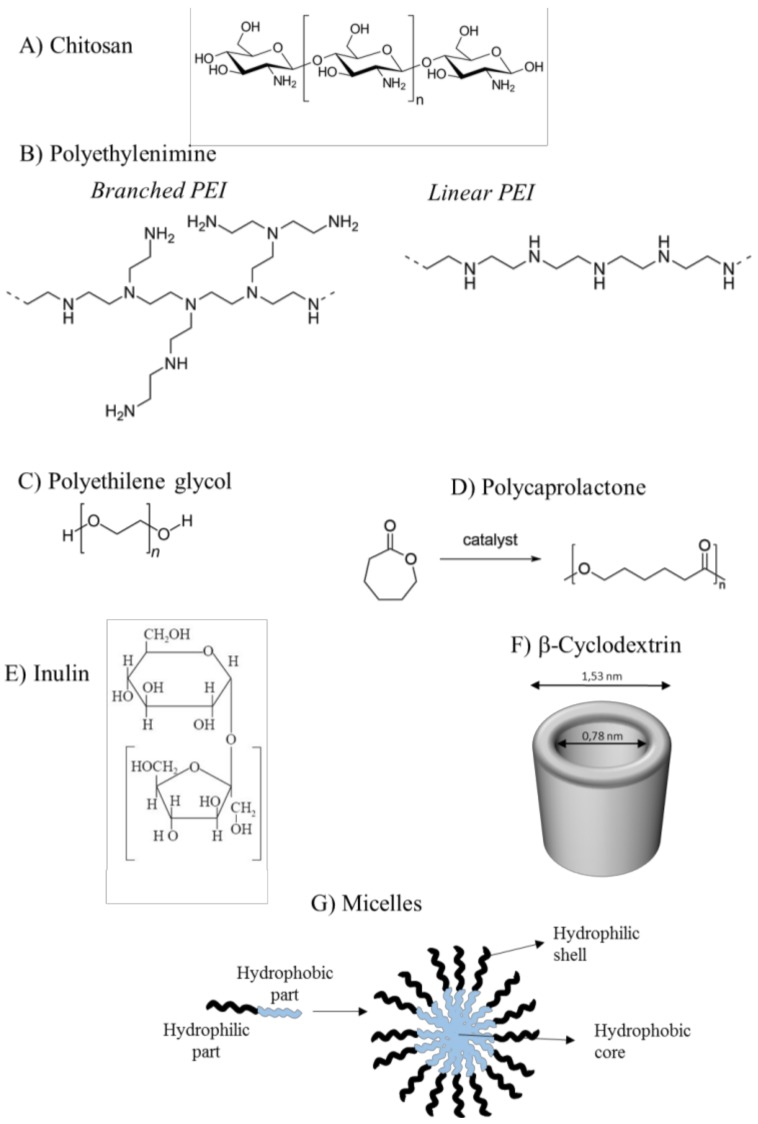
Polymers employed as siRNA delivery materials: chemical structures.

CH is produced commercially by deacetylation of chitin, the structural component present in the exoskeleton of crustaceans and cell walls of fungi. CH has been considered as a delivery material because of its unique physicochemical and biological characteristics [42]. It is a linear polysaccharide with a carbohydrate backbone containing two types of repeating residues, 2-amino-2-deoxy-glucose (glucosamine) and 2-*N*-acetyl-2-deoxy-glucose (N-glucosamine), linked by (1-4)-β-glycosidic linkage (Figure 3A). The primary hydroxyl and amine groups located on the backbone of CH allow chemical modification in order to control the physical properties. Moreover, the amino groups confer to CH a positive charge. This favors the electrostatic binding with siRNAs, which, containing phosphate groups in their structure, are negatively charged. Thus, CH can confer protection to siRNA driving it to the target cells. However, CH tends to suffer from the low transfection efficiency and low solubility. The conjugation with other molecules such as PEI and PEG can easily circumvent the problem. Alternatively, the physicochemical and biological properties of CH can be tuned by varying the degree of the acetylation and/or modifying the molecular weight [43]. 

PEI is a synthetic polymer with repeated units composed of amine groups and two carbon aliphatic CH_2_–CH_2_ spacer (Figure 3B). PEI can exist both in linear or branched forms. In the linear form, it contains all secondary amines; in contrast, branched PEI contains primary, secondary and tertiary amino groups. PEI is produced on industrial scale and finds many applications mostly due to its poly-cationic nature. Even if PEI tends to be more cytotoxic than natural polymers [44], it is one of the most used cationic polymers in the bio-medical field. The ability to transfer nucleic acids to the cell is superior for high molecular weight PEI compared to low molecular weight PEI. However, high molecular weight PEI displays somewhat more cytotoxic effects compared to low molecular weight PEI. The addition of hydrophilic and hydrophobic segments or cell/tissue-specific ligands [45] can however significantly reduce cytotoxicity. Because of the positively charged amino-groups, PEI can efficiently bind negatively charged siRNAs. Additionally, due to its “proton sponge effect”, PEI favors siRNA endosomal escape [46].

PEG is a synthetic polyether compound also known as polyethylene oxide (PEO) or polyoxyethylene (POE), depending on its molecular weight. Commonly, its structure is represented by the formula H−(O−CH_2_−CH_2_)n−OH (Figure 3C). PEG, considered to be non-toxic and safe [47], is widely used due to the solubility in an aqueous environment and organic solvents. PEG addition (PEGylation) reduces toxicity and stabilizes the delivery particles [48]. PEG can be also used as anchor for specific ligands to be fixed on the surface of delivery particles [49]. 

PCL is a polyester of synthetic origin [50], produced by the ring opening polymerization of the cyclic monomer ε-caprolactone in the presence of a catalyst (Figure 3D). PCL is biodegraded via the hydrolysis of its ester linkages in physiological conditions. Once biodegraded, it is fully excreted from the body. For this reason, it received a great deal of attention as an implantable/drug delivery biomaterial, applications for which FDA has approved it. Additionally, PCL can be used to tune the physical, chemical and mechanical properties of different materials when co-polymerized, for example, with polymers such as PEG.

Inulin is a natural storage carbohydrate present in many plant species [51]. Fructose units, joined by a β(2→1) glycosidic bond, with a terminal glucose (Figure 3E) compose the polymer. Inulin is used in many food applications and more recently in pharmaceutical applications. For example, inulin can be used to improve the water solubility of various poorly soluble drugs. Additionally, chemically modified inulin can be used in the controlled delivery to the colon. This is possible as inulin is hydrolyzed only by inulinases produced by bifidobacteria in the colon and not by the digestive enzymes in the upperparts of the gastro intestinal tract. We have recently shown that inulin conjugated with diethylenetriamine is suitable as a carrier for therapeutic delivery of siRNAs [52].

Cyclodextrins (CDs) are a series of natural cyclic oligosaccharides composed of 6, 7 or 8 d-glucose units linked by α-1,4-linkages and named α-, β-, or γ-CD. CDs are characterized by a truncated cone shape with the internal cavity being hydrophobic (Figure 3F) while the outer part is hydrophilic. The cavity depth depends on the CD type being around 0.7 nm, 0.78 and 0.9 for α-CD, β-CD and γ-CD, respectively. This cavity can host various molecules to form supra-molecular inclusion complexes. The most commonly used form of CD is β-CD that is equipped with amphiphilic and cationic residues. The presence of positively charged residues allows the interaction with the negatively charged siRNAs. CD are commonly inserted in polymeric structures such as a linear or branched polymer thus resulting in delivery vectors characterized by low cytotoxicity and effective transfection ability [53].

From the examples above reported it is evident that electrostatic interaction are at the base of the interaction between cationic copolymers. Following binding to the polymers, siRNA half-life and cell entry are greatly favored. For siRNA delivery, polymers can be also used in the form of more complex structures named micelles. Single units (unimers) make up these structures. The hydrophilic portion of the unimers is in contact with the external aqueous environment while the hydrophobic one stays in the inner part of the micelles (Figure 3G) [4]. To obtain unimers with such an amphipathic property polymers are conjugated with, for example, stearic acid (SA), a saturated fatty acid with an 18-carbon chain. This kind of modification can thus confer a great plasticity to polymers [54]. Other cationic polymers able to form micelles in solution are PEG and poly(propylene sulfide). Example of hydrophobic polymers are PCL, polylactide (PLA) and poly(lactic-co-glycolic acid) (PLGA).

## 4. Specific Strategies to Optimize siRNA Delivery to HCC

**Figure 4:**
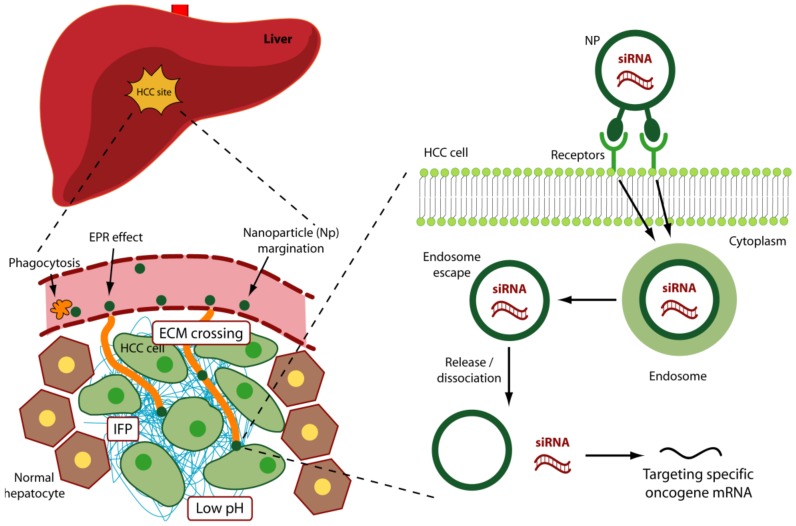
Specific aspects related to an optimized siRNA delivery to HCC.

In addition to the above-reported strategies useful to improve in general siRNA delivery, other specific aspects should be considered for siRNA delivery to HCC cells. In particular, vascular, physical and molecular aspects need consideration (Figure 4).

### 4.1. Vascular Aspects

Whereas HCC is mostly nourished by arterial vessels, the normal liver is mainly supplied by vein vessels [55]. Notably, the arterial supply in HCC increases with the progression of the disease being moderate in initial lesions and becoming preponderant in advanced HCC [56]. This shift occurs to meet the higher requirement of nutrition and oxygen of bigger and advanced HCC lesions. Thus, to target HCC and preserve the normal liver tissue, it would be desirable to consider an arterial delivery for siRNA in the advanced stages of the disease. In this regard, the use of TACE [57] can be considered an appropriate approach for siRNA delivery. TACE is a minimally invasive procedure performed in interventional radiology to restrict the HCC blood supply. Small embolic particles coated with chemotherapeutic agents are injected selectively into an artery directly supplying the tumor thus targeting mostly HCC cells but not normal hepatocytes. In this regard, in animal models of HCC, trans arterial drug delivery resulted: (1) to be safe both for the liver and for distant organs, (2) to display preferential tumor uptake of the anti-cancer drug and (3) to have high tumor response [58]. With regard to the delivery of siRNA, an anti hypoxia-inducible factor-1α (HIF-1α-siHIF-1α) was released by a trans arterial embolization procedure complexed within a iodized oil emulsion [59]. HIF-1α, a pivotal transcription factor synthesized by tumor cells under hypoxic conditions, regulates the expression of relevant genes for the survival of cancer cells. The combination of trans arterial embolization with siHIF-1α resulted to be effective in slowing down tumor progression and improving animal survival. 

In addition to the dissimilar vascularization, HCC differs from the normal liver because of the different structure of the nourishing vessels. HCC vessels are characterized by an unusual leakiness, [60], giving origin to the well known as enhanced permeability and retention (EPR) effect [61]. This is due to the presence of intercellular fenestrae in the vessel wall of about 1.7 μm [62]. Additionally, the frequent absence of pericytes and/or vascular smooth muscle cells around the endothelial layer, contributes to the leakiness of the tumor vessels. Thus, HCC vessels are more prone to allow the extravasation of particles around 1 μm in diameter compared to the normal liver vessel where fenestrae size is significantly smaller. Additionally, it has been reported that particle of about 1–3 μm in diameter [63] are mostly confined in proximity of the endothelial cells of the vessel; in contrast, smaller particles tend to stay more in the middle of the vessel. Thus, extravasation is favored for 1–3 μm particles compared to smaller particles. Moreover, μm size particles are not removed by the kidney, which has a smaller cut off (8 nm) [64,65].

### 4.2. Phagocytosis

Despite the above advantage of the 1–3 μm particles, it should be reminded that particles bigger then 0.2 μm are readily scavenged non-specifically by macrophages [66]. In the liver, phagocytosis, mostly exerted by Kupffer cells, is particularly increased due to the reduced blood flow (200–800 μm·s^−1^ compared to 10–100 cm·s^−1^ in veins [67] in hepatic sinusoid compared to other body vessels. 

The charge of the nanoparticle surface can also influence phagocytosis. In general, nanoparticles with highly positive or negative charges are more prone to phagocytosis due to serum protein absorption [68]. To minimize the uptake by the liver cells different form HCC cells, PEGylation is often followed. PEGylation reduces nanoparticle binding to serum protein (such as immunoglobulin) thus impairing macrophage uptake. However, it should be considered that also nanoparticle shape plays a role in phagocytosis. It has been shown that while spherical nanoparticles undergo phagocytosis, disc-like and cylindrical nanoparticles more successfully evade phagocytosis [69]. Reducing serum protein binding to nanoparticles has also the advantage to minimize unwanted variation in size, surface charge and aggregation of the nanoparticles. Serum protein can indeed significantly affect nanoparticle fate. Albumin can prolong blood nanoparticle circulation [70] and displace negatively charged molecules such as siRNAs. On the other hand, binding to the positively charged fibrinogen can alter the surface charge and improve phagocytosis [71]. 

Finally, it should be considered that all the above aspects are particularly problematic when administering the nanoparticles systemically; however, a local administration via TACE, feasible in HCC, can significantly minimize most of them. 

### 4.3. Physical Aspects

As in most other tumors, defective neo-vasculature of HCC determines ineffective oxygen delivery, especially in the inner regions of the tumor. This implies that HCC cells can obtain their energy mostly from the biochemical pathway that can work in low oxygen, i.e., glycolysis [72]. The increased glycolytic rate is invariably associated with the increased production of lactic acid. This, together with the reduced H^+^ removal by the defective neo-vasculature, determines a reduction in pH within the tumor. Thus, it may be possible to take advantage of this feature by creating siRNA delivery systems able to preferentially release siRNA in low pH [73].

Another physical aspect to be considered for effective siRNA delivery is the interstitial fluid pressure (IFP). The liquid phase present between the newly formed tumor vascular wall and the plasma membrane of the neoplastic cells is responsible for the IFP phenomenon [74]. IFP is higher in the center of the tumor rapidly diminishing towards the tumor periphery. The reasons for IFP are multiple and include vessel abnormalities, diminished function of lymphatic vessels [75] and the EPR effect [76]. The occurrence of this center-periphery gradient of the liquid pressure in many tumors including HCC [74], determines a mass flow movement of fluid away from the central region of tumor. This phenomenon obviously reduces the amount of drug (including siRNA) that can reach the inner part of the tumor. On the other hand, moving to the inner part, the amount of necrotic cells increases due to the low oxygen concertation. Thus, it may be not so crucial to reach necrotic cells; in contrast, reaching the vital tumor cells closer to the periphery may be more relevant.

The extracellular matrix (ECM) of many tumors tend to be denser than in the normal corresponding tissue. This is mostly due to the deregulation of ECM protein/glycoprotein/proteoglycan interaction [77]. A possibility to minimize the shielding effect of tumor-specific ECM is to minimize particle size and/or equip the particles with ECM proteases [78]. It should be noted however, that in the case of HCC, ECM is not particularly dense [79] and thus this barrier may not represent an insurmountable obstacle. Despite this, further investigation about the physical characteristic of HCC ECM are required (work in progress).

### 4.4. Molecular Aspects: Targeting Surface Antigens of HCC Cells

Optimal HCC delivery systems for siRNA should take into account the above aspects. However, this might not be sufficient for the development of an effective and specific delivery approach for HCC. The generation of delivery systems equipped with smart moieties able to recognize specific antigens on HCC cells is considered extremely relevant. This strategy can significantly contribute to restrict the delivery of therapeutic molecules to HCC cells, leaving the normal hepatocyte un-affected.

Different molecules predominantly expressed by HCC cells compared to normal hepatocytes have been considered so far as possible targets (Table 1). Among this, Asialoglycoprotein receptor (ASGP-R) is a glycoprotein mostly presents on the hepatic cell surface [80]. Notably, it is overexpressed on the surface of HCC cells [81]. ASGP-R is capable of recognizing and binding galactose terminal and galactosamines residues. Because of its restricted expression, ASGP-R is considered a valuable surface biomarker to target HCC cells [82].

Another target is Glypican-3 (GPC3), a member of membrane-bound heparin sulfate proteoglycans. Nowadays GPC3 is considered a highly specific marker for HCC [83]. However, its expression is not limited to HCC as it is present also on other tumor cells such as squamous cell carcinoma of the lung and testicular non seminomatous germ cell tumors [84]. 

Transferrin receptor (TfR), a transmembrane glycoprotein, is involved in the uptake of the iron-carrying protein named transferrin [85]. Whereas TfR is poorly expressed by normal cells, in proliferating cells its expression rises significantly [86]. Moreover, TfR-2, a newly identified member of TfR family, was found to be preferentially expressed in hepatocytes and enterocytes of the small intestine [87]. Thus, TfR-2 better than TfR, may be considered a specific ligand to target hepatocytes.

The folic acid (FA) receptor (FR) is a 38 kDa glycosyl-phosphatidylinositol membrane-anchored glycoprotein. Whereas it is overexpressed in various cancers, its expression in normal tissues is considerably reduced compared to tumor tissues. This distinct expression pattern makes it an ideal target for tumor drug delivery. Moreover, it has been extensively studied in targeted drug delivery due to its inherent high affinity and small size [88]. 

Epidermal growth factor receptor (EGFR), also known as ErbB1 or HER-1, is a classical transmembrane receptor tyrosine kinase. It is activated by several ligands, which in turn trigger different pathways controlling proliferation, differentiation and survival. Notably, human carcinomas frequently express high levels of receptors in the EGFR receptor family, and their overexpression has been associated with a more aggressive clinical behavior [89].

Integrins are transmembrane receptors involved in cell-ECM adhesion. Upon ligand binding, integrins activate signal transduction pathways that regulate many relevant cellular processes such as cell proliferation. Notably, the αvβ3 and αvβ5 integrins are overexpressed on the angiogenic endothelium in different malignant tumors [90] including HCC. Thus, by equipping a delivery system with the tripeptide arginine glycine aspartic acid (RGD) motif that is bound by αvβ3/αvβ5 integrins, it may be possible to direct therapeutic siRNAs to HCC.

The scavenger receptor class B type I (SR-BI receptor) is a multi-ligand cell surface receptor expressed on both macrophages and liver cells [91]. It facilitates the uptake of cholesteryl esters from HDL. Thus, it drives cholesterol from tissues to the liver in the various stages of reverse cholesterol transport pathway. By preparing a delivery vector able to target SR-BI receptor, it may be possible to gain HCC targeting. 

Despite being in principle of interest for liver targeting, the above mentioned molecules are mostly expressed on both normal liver and HCC cells, although at different levels. Thus, a real discrimination between healthy and HCC cells is not fully reachable. This limitation does not seem to apply for the homodimeric glycoprotein (AF-20 antigen) which is expressed on HCC but not on normal liver cells [92]. A completely novel approach may be based on the use of specific aptamer binding to unknown HCC specific antigen. Aptamers are represented by single-stranded sequences of DNA or RNA with the ability to specifically interact with a target molecule. The specific aptamer folding is responsible for the possibility to link the target molecule; thus, almost no Watson-Crick base pairing occurs between the aptamer and the target. The fact that aptamers are small, easy to select, can be directed against any target, are cheap to produce, are stable and are not immunogenic [93], confers to these molecules a great advantage compared to antibodies. To date, an aptamer (TLS11a) has been shown [94] to bind HCC cells specifically. The effectiveness of this approach needs further confirmation.

### 4.5. Molecular Aspects: Targeting HCC Specific Oncogenes

The above discussion indicates that ideal HCC markers have not yet unequivocally identified. Indeed, most of them are also present on normal liver cells. Thus, a complete discrimination between HCC cells and normal hepatocytes cannot be guaranteed. However, an increased level of targeting specificity can be achieved using siRNA directed against cellular genes/pathway expressed/activated predominantly in HCC cells. This implies that, even if a siRNA reaches a healthy hepatocyte, it may not exert major detrimental effects as the target gene is not/poorly expressed. Below, some possible examples of HCC specific oncogenes are discussed.

eEF1A, a protein involved in the elongation step of protein synthesis [95], has two isoforms, eEF1A1 and eEF1A2. In addition to the role in translation, both eEF1A isoforms possess other functions relevant for many cellular processes such as cell cycle and apoptosis [96]. eEF1A1 is expressed in almost all human tissues; in contrast, eEF1A2 expression is confined to skeletal muscle, heart and nervous system with no expression in the normal liver. We reported that eEF1A2 is overexpressed in human HCC cell lines and that its overexpression correlates with cancer cell growth and differentiation phenotype [97]. eEF1A2 overexpression has been also detected in HCC human tumor specimens [98,99] and its role in HCC development has been proved [100]. The fact that eEF1A2 is not expressed in the normal liver but its expression is pathologically turned on during HCC development, suggests that an anti eEF1A2 siRNA may exert its effect in HCC cells but not in normal hepatocytes. Thus, even if the delivery vector cannot perfectly discriminate between HCC and normal hepatocytes, detrimental effects on healthy hepatocytes are not expected due to the lack of eEF1A2 expression. 

E2 promoter binding factor 1 (E2F1) [101], belongs to a family of transcription factors named E2Fs [102]. E2F1 is a transcription factor implicated in the regulation of many cellular processes including cell migration, differentiation, metabolism, cell reprogramming and proliferation. With regard to cell proliferation, E2F1 triggers the G1/S phase transition of the cell cycle by promoting the expression of genes such as cyclin E and cyclin A. Thus, E2F1 is considered a pivotal regulator of the initial stages of cell proliferation. Whereas its expression is ubiquitous in human tissues, the biological effects are somewhat tissue-dependent. It has been reported that E2F1over-expression does not confer any growth advantage to hepatocytes following surgical elimination of two-thirds of the liver [103]. Additionally, it has been [104] suggested that another E2F family member, E2F-4, rather than E2F1 is critical for liver cell growth. In contrast to the normal liver, E2F1 plays a documented role in HCC development [102] where it is overexpressed. Due to the E2F1 features, it is reasonable to speculate that its siRNA-mediated silencing may predominantly affect the growth of HCC cells leaving normal hepatocytes substantially unaffected. 

Whereas eEF1A2 and E2F1 are not the only smart targets for HCC as other may exist [105], it is clear that a double level of targeting specificity should be pursued, i.e., the use of targeting moieties on the delivery system and the employment of siRNA directed against the mRNA of specific HCC genes/pathways. 

### 4.6. Description of siRNA Activity by Mathematical Modeling

Based on all the above considerations, the major steps for nanoparticle delivery to HCC are: circulation in the blood, accumulation in the tumor area, internalization into tumor cells followed by the siRNA release [106]. Whereas these variables can be investigated independently at the experimental level, for a comprehensive determination of the delivery effectiveness, mathematical modeling can be of great value. Although the mathematical description of such a complex phenomenon is not an easy task, it can provide important indication on how to optimize delivery by reducing the time-consuming and expensive experimental part. In this regard, the model built up by Bartlett and Davis [107] represents a powerful theoretical tool to describe siRNA-complex fate, once injected in the blood stream. Indeed, these authors recur to a set of twelve ordinary differential equations aimed to estimate the temporal evolution of the concentration of twelve very important quantities. These are the plasma free (*SC*) and bound (to plasma protein, *SC_B_*) siRNA-complex concentration, siRNA-complex concentration in the extracellular environment close to target cells (*SC_T_*), siRNA-complex and free siRNA concentration inside cell endosomes and cytoplasm (*SC_E_*, *S_E_*, *SC_C_* and *S_C_*, respectively), activated RISC (RNA-induced silencing complex) complex concentration free and bound to target mRNA (RC* and *R_m_*, respectively), target mRNA (*C_mRNA_*), protein (*Pr*) concentration and cells number (*Nc*). This model is organized into four modules that can be changed independently to modify model complexity as desired. The first module deals with siRNA “circulation/extracellular transport” (three equations), the second is about siRNA “cellular uptake and intracellular trafficking” (four equations), the third describes the fate of free and bound activated RISC (three equations) according to the Micaelis-Menten multiple turnover mechanism and the fourth focusses on “cell growth and target protein production” (two equations):

Circulation/extracellular transport:

(1) $$\frac{dSC_{B}}{dt}=K_{B}^{P}SC-K_{U}^{P}SC_{b}$$

(2) $$\frac{dSC}{dt}=K_{U}^{P}SC_{B}-\left(K_{B}^{P}+K_{EL}^{P}+X_{sc}K_{t}\right)SC$$

(3) $$\frac{dSC_{T}}{dt}=X_{sc}K_{t}SC-\left(K_{EL}^{T}+K_{int}\right)SC_{T}$$

Cellular uptake and intracellular trafficking:

(4) $$\frac{dSC_{E}}{dt}=K_{int}\frac{V_{E}}{V_{I}}SC_{T}-\left(dil+K_{U}^{E}+K_{esc}^{E}\right)SC_{E}$$

(5) $$\frac{dS_{E}}{dt}=K_{U}^{E}SC_{E}-\left(dil+K_{S-d}^{E}+K_{S-esc}^{E}\right)S_{E}$$

(6) $$\frac{dSC_{C}}{dt}=K_{esc}^{E}SC_{E}-\left(dil+K_{U}^{C}\right)SC_{C}$$

(7) $$\frac{dS_{C}}{dt}=K_{U}^{C}SC_{C}+K_{S-esc}^{E}S_{E}+K_{RISK-b}^{C}RC^{*}-\left(dil+K_{S-d}^{C}\right)S_{C}-K_{RISK-f}^{C}S_{C}\left(R_{tot}-RC^{*}-R_{m}\right)$$

Micaelis-Menten multiple turnover mechanism:

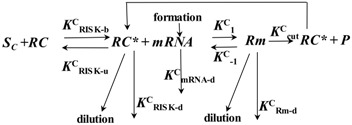


Cell growth and target protein production:

(8) $$\frac{dPr}{dt}=K_{f}^{Pr}C_{mRNA}-K_{d}^{Pr}P_{r}$$

(9) $$\frac{dNc}{dt}=K_{g}N_{c}\left(1-\frac{N_{c}}{N_{cmax}}\right)$$

where *t* is time, $K_{B}^{P}$, $K_{U}^{P}$, $K_{EL}^{P}$ are, respectively the binding, unbinding and elimination siRNA-complex constants in the plasma environment, X_sc_ is the effective dose fraction available to target cells, *K*_t_ is a kinetic transfer constant affecting siRNA-complex flux to tissues close to target cells, *K*_int_ is the internalization constant inside target cells, $K_{EL}^{T}$ is the elimination siRNA-complex constant in the tissues close to target cells, *V*_E_ and *V*_I_ are, respectively, extracellular volume and intracellular volume, *dil* is the dilution rate due to cells division, $K_{U}^{E}$ and $K_{U}^{C}$ are, respectively, the siRNA-complex unbinding constants inside endosomes and cytoplasm, $K_{esc}^{E}$ and $K_{S-esc}^{E}$ are, respectively, the kinetic constant ruling siRNA-complex and free siRNA transfer from endosomes to cytoplasm, $K_{S-d}^{E}$ and $K_{S-d}^{C}$ are, respectively, the free siRNA degradation constant inside endosomes and cytoplasm, $K_{RISK-f}^{C}$ and $K_{RISK-b}^{C}$ are, respectively, the forward and backward kinetic constants ruling RISK activation, *R*_tot_ is the total concentration of RISC complex, $K_{1}^{C}$, $K_{-1}^{C}$ and $K_{cut}^{C}$ are the Micaelis-Menten kinetic constants, $K_{RISK-d}^{C}$, $K_{mRNA-d}^{C}$ and $K_{Rm-d}^{C}$ are, respectively, the activated RISK, mRNA and Rm degradation constants inside cytoplasm, $K_{f}^{Pr}$.and $K_{d}^{Pr}$ are two kinetics constants ruling the protein concentration time dependence, *K_g_* is a constant ruling the cells number time variation and *N_cmax_* is the maximum admissible cells number. 

Interestingly, from a physical point of view, the delivery process can be subdivided into two distinct phases: the external one, involving the first module, and the cellular one, involving the other three modules. Thus, in the case of in vivo experiments, all the four modules are needed, while only the last three modules matter in the case of simpler in vitro experiments.

**Figure 5:**
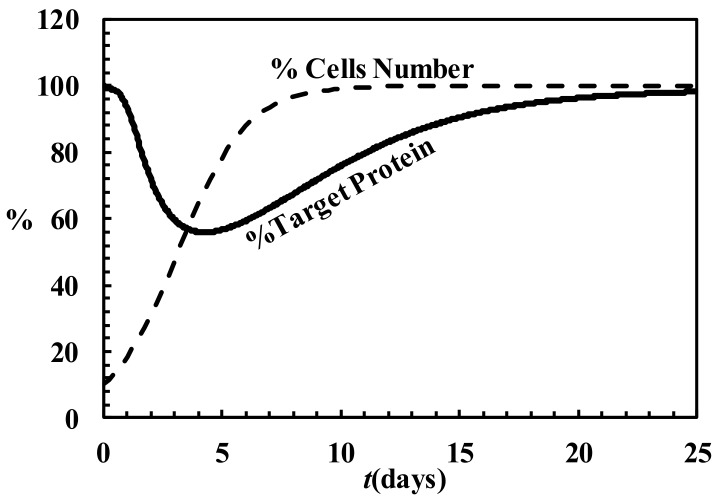
Temporary evolution of the % of target protein (solid line) and the % of cells number (dashed line) according to the Bartlett & Davis model (model parameters are those reported in Bartlett & Davis [107]).

One of the major merit of this model relies on the possibility of evaluating the timely evolution of the target RNA and protein both in in vitro and in vivo experiments. This is a very important achievement as it allows comparing theoretical predictions with experimental data. In turn, this model permits the estimation of many parameters having a precise scientific meaning such as the Micaelis-Menten constants. Figure 5 shows, as an example, the temporary trend of the target protein (expressed as the % of the initial value) and the number of cells (expressed as the % of the maximum number of cells) after a single administration of siRNA-complex. It is clear that the effect of siRNA-complex is to initially reduce the amount of the target protein whose concentration reaches a minimum after, about, four days. Then, due to siRNA-complex degradation and the continuous dilution due to cell growth, the amount of the target protein comes back to its original value.

The aspect of the model that needs to be implemented deals with the evaluation of siRNA-complex dilution due to cell replication. Indeed, the assumption of a constant dilution does not seem realistic as dilution depends on the number of cells that, in turn, relies also on the effect exerted by siRNA on the target RNA/protein whose abundance reflects the cell replication speed. Thus, we are working on a model implementation accounting for an intrinsically variable dilution. Despite this, it is evident that the mathematical modeling of the entire process can contribute to better understand the phenomenon and, especially, to guide the experiments.

## 5. Strategies Utilized to Deliver siRNA to HCC 

Among the several studies aimed at the delivery of therapeutic siRNAs to HCC, here we will concentrate on the strategies based on targeted systems (Table 2 and Table 3).

### 5.1. In Vitro Models of HCC 

Lu Han et al. [108] developed a multifunctional vector for oral administration consisting of galactose modified trimethil chitosan-cysteine (GTC) conjugates. The galactose residue allowed the targeting towards ASGP-R, overexpressed on HCC cells [80,81,82]. Three different GTC nanoparticles (NPs) with various galactose grafting densities were tested. NPs were loaded with anti-Survivin and anti-VEGF (Vascular Endothelial Growth Factor) siRNAs. Survivin, transcribed from the BIRC gene, is a protein overexpressed in most human tumors but not in normal tissues [109]. It prevents apoptotic cell death through the inhibition of caspase activation. VEGF increases the protein level of Survivin via the activation of PI3K/AKT pathway and promotes tumor angiogenesis [110]. The ability of the NPs to cross the intestinal epithelium was assessed in vitro both in Caco2 cells and in Caco2 cells co-cultured with Raji B lymphocytes. Caco2 cell line has been used to mimic the intestine epithelium; Raji B lymphocytes mimic the lymphatic M cells of the intestine wall. The GTC NPs reversibly open the tight junctions between Caco2 and Raji B lymphocytes cells, thus promoting transcytosis of NPs. This suggests that in vivo, the NPs can have the ability to cross the intestinal wall. In the HCC cell line BEL-7402, the most effective NPs displayed 1,9-fold increased uptake as compared to the control, indicating the effectiveness of ASGP-R targeting by the galactose residue. Once internalized, the inhibition of cell growth exerted by NPs loaded with both anti-Survivin and anti-VEGF siRNAs was more pronounced than that induced by a single siRNA, indicating the synergistic antitumor effect. 

Huang et al. [111] suggested a multifunctional polymeric micelle composed of PEI grafted with stearic acid (PEI-SA). Moreover, the particles contained folic acid-polyethyleneglycol-block-polyglutamic acid to improve cellular uptake. The targeting moiety was represented by FA (folic acid), which can drive the complex to the FR (folic acid receptor), typically overexpressed in tumor cells. The nanoparticles were used for the co-delivery of anti-VEGF siRNA and doxorubicin (DOX), a commonly used anticancer drug. The nanoparticles display positively charged amino groups, which enable the electrostatic interaction with the negatively charged phosphate of the siRNA. The safty, cellular uptake and specific VEGF gene silencing ability were demonstrated in the HCC cell line HuH-7 cells. 

Wang et al. [112] synthetized a micellar nano-carrier containing PEG and PCL polymers. In this case, a *N*-acetylgalactosamine functionalized PEG-b-PCL (GalNac- PEG-b-PCL) and PCL-b-PPEEA (poly(2-aminoethyl ethylene phosphate), were used. The first polymer, carrying *N*-acetylgalactosamine, conferred the ability to bind to ASPG-R, thus allowing a liver targeted delivery. The second allowed the electrostatic binding to the siRNA that was directed against apolipoprotein B. This is a liver-specific cholesterol transport-related gene, chosen as a model target that has no directed correlation to HCC. This study confirmed the effectiveness of the micellar di-block polymers nano-carrier approach in vitro.

We recently developed a siRNA delivery system based on inulin (Inu), an abundant and natural polysaccharide, conjugated with diethylenetriamine (DETA) residues (Inu-DETA) [52]. Inu-DETA polyplexes can effectively bind siRNAs, are highly cyto-compatible and, in the HCC cells JHH6, effectively deliver a siRNA directed against the mRNA of the transcription factor E2F1. In JHH6, E2F1 silencing resulted in a marked anti-proliferative effect. We also observed that in JHH6, the mechanisms of polymer-siRNA uptake and trafficking, i.e., micropinocytosis and clathrin mediated endocytosis, is crucial to allow the proper siRNA cellular distribution and thus effectiveness. In contrast, in the bronchial cell line 16HBE where the uptake mechanism (caveolae-mediated endocytosis) resulted in an improper distribution of siRNA within the cell, no significant effects were observed. These data point towards the relevance of the proper trafficking for efficient siRNA delivery. Moreover, they suggest that delivery specificity may be also achieved at the trafficking level. It should be noted that our Inu-DETA nanoparticles had no specific HCC targeting moieties but reached specificity of action with a different strategy. Additionally, the targeting of E2F1 can confer a further level of specificity for HCC, as discussed in Section 4.5. Finally, it is obvious that a complete targeting specificity cannot be achieved only at the trafficking levels, as other targeting strategies have to be combined.

**Figure 6:**
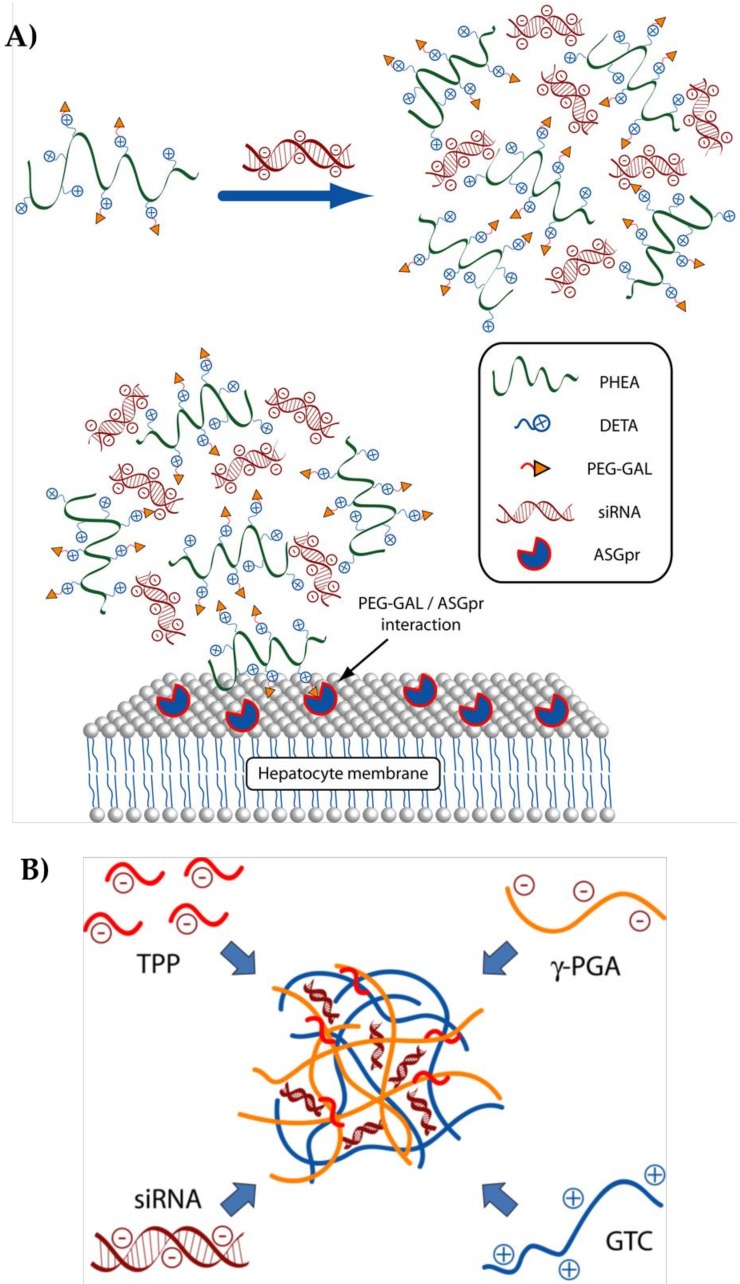
(**A**) Schematic representation of the polymer from ref. [113]; PHEA: α,β-poly-(*N*-2-hydroxyethyl)-d,l-aspartamide, DETA: diethylene triamine, PEG: polyethylene glycol, GAL galactose, siRNA: small interfering RNA, ASGP-R: Asialoglycoprotein receptor; (**B**) Schematic representation of the polymer from ref. [114]. GTC: galactose modified trimethyl chitosan-cysteine, TPP: sodium tripolyphosphate, γ-PGA: γ-polyglutamic acid; on the left are shown the single components, on the right the assembled particle.

We also concentrated on the development of a delivery system able to recognize a specific HCC cell antigen [113]. We prepared a cationic polymer based on α,β-poly-(*N*-2-hydroxyethyl)-d,l-aspartamide (PHEA) derivatized with diethylene triamine (DETA) and with a polyethylene glycol (PEG) derivative bearing galactose (GAL) molecules (PHEA-DETA-PEG-GAL) (see also Figure 6A). A cartoon of the polymer is reported in Figure 6A. The generated polymer had the proper features to be potentially employed as a systemic delivery system for siRNA. Moreover, it could specifically release an anti E2F1 siRNA to HCC cells. This property was due to the existence of GAL residues that can interact with the ASGP-R, overexpressed on HCC cells. Notably, our polymer was effective in reducing the mRNA and protein levels of E2F1 and of E2F1-regulated genes in ASGP-R overexpressing but not in ASGP-R non-expressing cells. In turn, a significant reduction in HCC cell proliferation was achieved. Notably, removal of GAL residue almost completely abrogated the targeting specificity of the developed polyplexes. Similarly, in ASGP-R non-overexpressing cells, polyplexes could not deliver the siRNA.

### 5.2. In Vivo Models of HCC 

In a recent study [114], nanoparticles containing urocanic acid-modified galactosylated trimethyl chitosan (UA-GT) conjugates as polymeric vectors were prepared. These nanoparticles displayed potent capabilities in encapsulating a siRNA against VEGF (siVEGF). The presence of the galactose residue allowed a specific uptake in the HCC cell line QGY-7703 via the binding to ASGP-R. Moreover, the urocanic acid residues improved the buffering capacity thus allowing a more efficient release from endosomes. For in vivo testing, a HCC mouse xenograft subcutaneous model of HCC was used. 200 µg of siVEGF/kg were administered systemically once every four days for a total of five times. siVEGF loaded nanoparticles significantly reduced tumor growth and significantly decreased the density of blood vessels demonstrating the anti angiogenic effects of the nanoparticles. Notably, the authors did not observe a significant loss in body weight and histological damage in the liver and other major organs. In line with this, the hematologic and hepatic function parameters were not significantly affected. Together, the above data suggest that the UA-GT nanoparticles were effective and potentially safe for in vivo use. 

Nanoparticles containing galactose-modified trimethyl chitosan-cysteine (GTC) conjugates [108] were used for the active targeting of ASGP-R. Nanoparticles loaded by anti-Survivin and anti VEGF siRNAs, were administered orally in a xenograft mice model of HCC. Trimethyl and cysteine groups of GTCs improved intestinal adhesion also promoting tight junctions opening. The galactose ligands allowed active internalization into HCC cells. Tumor growth rate was evidently reduced with no relevant cytotoxicity. In a variation of the above experiment, a series of GTCs polyplexes with distinct siRNA binding affinity were produced by modifying the composition of different ionic crosslinkers such as sodium tripolyphosphate (TPP) and γ-polyglutamic acid (γ-PGA) [115]. A cartoon of the GTC/TPP/γ-PGA polymer is reported in Figure 6B. In this case, only an anti VEGF siRNA was loaded. In xenograft mouse models of HCC, GTC polyplexes demonstrated to be safe and to significantly down regulate tumor angiogenesis causing a remarkable tumor growth arrest. 

In a recent study [116], a magnetic resonance imaging (MRI)-visible vector that incorporated FA-modified SPION (superparamagnetic iron-oxide nanoparticles) for the targeting to FR was developed. SPION, composed by iron-oxide in the form of maghemite (γ-Fe2O3), are nanoparticles with low toxicity as they are contained in ferritin, the physiological storing protein of iron in the human body. They have the property of being superparamagnetic, which means that in the presence of an external magnetic field, they become magnetized and can be entrapped/visualized into the region where the magnetic field is applied. PEG and PEI were used to allow FA and siRNA binding to the nanoparticles thus resulting in FA-PEG-g-PEI-SPION complexes. The mRNA of the transducin beta-like 1 X-linked receptor 1 (TBLR1) was chosen as siRNA target. This is an important regulator of HCC proliferation and tumor angiogenesis [116,117]. In different mice models of HCC (orthotropic and xenograft models), this delivery system resulted in an active and specific interaction with hepatocytes through the FA-targeting and demonstrated to be a safe approach, easily trackable with non-invasive imaging tools [116].

The αvβ3 and αvβ5 integrins are overexpressed on the surface of the angiogenic endothelium of different malignant tumors including HCC [118]. RGD-PEG-*g*-PEI-SPIONs delivery particles were developed and loaded with an anti-Survivin siRNA [119]. In a subcutaneous mouse model of HCC, RGD-PEG-*g*-PEI-SPIONs injected via the tail vein, efficiently and specifically inhibited tumor growth promoting tumor cell apoptosis/necrosis. However, no significant effect on tumor angiogenesis was observed. This leaves open the possibility that mechanisms, other than those predicted, might have contributed to the effects of the RGD-PEG-*g*-PEI-SPIONs particles. 

## 6. Conclusions

The works above reported clearly witness the efforts of the scientific community for the development of siRNA delivery systems in general and for HCC in particular. Because of the effectiveness of action, siRNAs have the potential to be used as bullets to hit the expression of disease-causing genes. However, their intrinsic fragility makes their use as naked molecules impossible. Thus, it is necessary to develop delivery systems able to confer protection and targeting HCC cells. 

Among the different delivery materials so far tested, polymers represent a very attracting class of materials. Because of their plasticity, biocompatibility and relative low costs, polymers are appealing materials, which can be employed to generate sophisticated and smart delivery systems. It is now clear that the delivery vector not only has to provide protection against siRNA degradation, but it also needs to allow overcoming some key steps in the delivery process. In particular, it has to favor accumulation in the tumor, targeting of tumor cells and then allow efficient siRNA release into the cell.

In the specific case of siRNA delivery to HCC, we believe the following aspects have to be addressed. First, as the arterial supply in HCC increases with the progression of the disease, it may be advisable to consider the arterial delivery route (TACE) in more advanced HCC forms and vein in early HCC forms. Second, to favor the margination of the particles thus promoting the EPR effect and liver accumulation, the particles diameter should be of about 1 µm. Third, following extravasation, particles may be able to undergo fragmentation originating nm particles. This size seems to be appropriate for ECM crossing even if, as above discussed, this barrier may not be particularly difficult to overcome in HCC. Fourth, targeting of HCC cells may be achieved equipping the delivery polymers with specific antigen. Fifth, siRNA delivery should be triggered by the low pH, originated by the biochemical pathways preferentially active in the tumor cell. At this step, the endosomal escape property of the delivery material is also relevant. Sixth, the use of siRNA directed against specific HCC oncogenes can further improve specificity and effectiveness. Finally, the possibility to mathematical modelling the entire process can certainly contribute to optimize and speed up the development of HCC delivery strategies.

From all the above considerations, it is clear that the solution of the problem is definitively not easy and necessitates the fine-tuning of different variables. Only the synergistic action of different competences spanning from physiology, molecular biology, biochemistry, medicine and engineering can succeed in this task. Despite this, the many encouraging experimental works presented in this review can pave the way to the identification of novel and efficient delivery solutions for siRNA to HCC.

## Figures and Tables

**Table 1:** Specific surface antigens on HCC cells.

Extended Name	Abbreviation	References
Asialoglycoprotein receptor	ASGP-R	[80,81,82]
Glypican-3	GPC3	[83,84]
Transferrin receptor	TfR	[85,86,87]
Folic acid receptor	FR	[88]
Epidermal growth factor receptor	EGFR	[89]
αvβ3 and αvβ5 integrins		[90]
Scavenger receptor class B type I	SR-BI receptor	[91]
Homodimeric glycoprotein	AF-20 antigen	[92]

**Table 2:** In vitro targeted delivery of siRNAs to HCC.

Delivery Material	HCC Targeting Antigen	HCC Model	siRNA mRNA Target	Reference
Galactose modified trimethylchitosan-cystein (GTC)	ASGP-R	BEL-7402	Survivin and VEGF	[108]
PEI grafted with stearic acid (PEI-SA)	FR	HuH-7	VEGF	[111]
GalNac- PEG-b-PCL and PCL-b-PPEEA	ASGP-R	Primary hepatocytes	apolipoprotein B	[112]
Inulin and diethylentriamine (Inu-DETA) on α,β-poly-(*N*-2-hydroxyethyl)-d,l-aspartamide (PHEA) and DETA and PEG) and GAL molecules (PHEA-DETA-PEG-GAL)	Trafficking specificity	JHH6	E2F1	[52]
	ASGP-R	JHH6	E2F1	[113]

**Table 3:** In vivo targeted delivery of siRNAs to HCC.

Delivery Material and Particle Size	HCC Targeting Antigen	HCC Model	siRNA mRNA Target	Reference
Urocanic acid-modified galactosylated trimethyl chitosan (UA-GT) 170 nm	ASGP-R	QGY-7703 and mouse xenograft subcutaneous model (systemic delivery)	VEGF	[115]
Galactose modified trymethil chitosan-cystein (GTC) 130–160 nm	ASGP-R	xenograft mice model of HCC (oral administration)	Survivin and VEGF	[108]
GTCs polyplexes with distinct siRNA binding affinity 135–170 nm	ASGP-R	QGY-7703 and xenograft mice model of HCC (intra-tumor injection)	VEGF	[114]
FA-PEG-*g*-PEI-SPION 60 nm	FR	orthotropic and xenograft models (systemic delivery)	TBLR1	[116]
RGD-PEG-*g*-PEI-SPION 122 nm	αvβ3 and αvβ5 integrins	Bel-7402 and mouse xenograft subcutaneous model (systemic delivery)	Survivin	[119]
